# Association between Air Pollution and Short-Term Outcome of ST-Segment Elevation Myocardial Infarction in a Tropical City, Kaohsiung, Taiwan

**DOI:** 10.3390/toxics11060541

**Published:** 2023-06-19

**Authors:** Jyun-Bin Huang, Kuo-Chen Huang, Ting-Min Hsieh, Chih-Min Tsai, Hao-Yi Hsiao, Chi-Yung Cheng, Fu-Jen Cheng

**Affiliations:** 1 Department of Emergency Medicine, Kaohsiung Municipal Feng Shan Hospital—Under The Management of Chang Gung Medical Foundation, Fengshan District, Kaohsiung 830, Taiwan; u9001135@gmail.com; 2 College of Medicine, Chang Gung University, No. 259, Wenhua 1st Road, Guishan District, Taoyuan City 333, Taiwan; bluescratch7@gmail.com (K.-C.H.); hs168hs168@gmail.com (T.-M.H.); tcmnor@cgmh.org.tw (C.-M.T.); haoyi222@hotmail.com (H.-Y.H.); 3 Department of Emergency Medicine, Kaohsiung Chang Gung Memorial Hospital, College of Medicine, Chang Gung University, Kaohsiung 833, Taiwan; 4 Division of Trauma, Department of Surgery, Kaohsiung Chang Gung Memorial Hospital, College of Medicine, Chang Gung University, Kaohsiung 833, Taiwan; 5 Department of Pediatrics, Kaohsiung Chang Gung Memorial Hospital, No. 123, Dapi Road, Niao-Sung District, Kaohsiung City 833, Taiwan; 6 Division of Cardiology, Department of Internal Medicine, Kaohsiung Chang Gung Memorial Hospital, College of Medicine, Chang Gung University, Kaohsiung 833, Taiwan

**Keywords:** ST-segment elevation myocardial infarction, STEMI, particulate matter, nitrogen dioxide, emergency department, air pollution

## Abstract

ST-segment elevation myocardial infarction (STEMI), one of the primary factors leading to global mortality, has been shown through epidemiological studies to have a relationship with short-term exposure to air pollutants; however, the association between air pollutants and the outcome of STEMI has not been well studied. The aim of this study was to estimate the impact of air pollutants on the outcomes of STEMI. Data on particulate matter <2.5 μm (PM_2.5_), <10 μm (PM_10_), nitrogen dioxide (NO_2_), and ozone (O_3_) at each of the 11 air monitoring stations in Kaohsiung City were collected between 1 January 2012 and 31 December 2017. Medical records of non-trauma patients aged > 20 years who had presented to the Emergency Department (ED) with a principal diagnosis of STEMI were extracted. The primary outcome measure was in-hospital mortality. After adjusting for potential confounders and meteorological variables, we found that an increase in the interquartile range (IQR) in NO_2_ was associated with an elevated risk of in-hospital mortality in patients with STEMI. Moreover, there was an observed higher risk of in-hospital mortality associated with an increase in the IQR of NO_2_ during the warm season, specifically in lag 3 (3 days prior to the onset, OR = 3.266; 95%CI: 1.203–8.864, *p =* 0.02). Conversely, an IQR increase in PM_10_ was associated with an increased risk of in-hospital mortality in patients with STEMI in lag 3 (OR = 2.792; 95%CI: 1.115–6.993, *p =* 0.028) during the cold season. Our study suggests that exposure to NO_2_ (during the warm season) and PM_10_ (during the cold season) may contribute to a higher risk of poor prognosis in patients with STEMI.

## 1. Introduction

Growing evidence indicates the health effects of air pollution, particularly on the respiratory and cardiovascular systems [1,2]. Epidemiological studies have demonstrated that short-term exposure to air pollutants, including particulate matter <2.5 μm (PM_2.5_), <10 μm (PM_10_), and nitrogen dioxide (NO_2_), is strongly correlated with the risk of myocardial infarction (MI) events, emergency department (ED) visits, and stroke [3,4,5]. Toxicological investigations have also unveiled that both short- and long-term exposure to air pollution may result in vascular dysfunction [6,7], lung inflammation [8], and even disturbances in blood pressure regulation [9]. 

According to the American Heart Association, a sudden decrease in blood flow in the coronary arteries is the primary trigger for ST-segment elevation myocardial infarction (STEMI), resulting in a significant contribution to the overall mortality rates worldwide [10]. Recent studies have shown a direct link between short-term exposure to air pollution, ED visits, hospitalization, and related mortality risks for STEMI [11,12]. However, lack of evidence focused on the impact of air pollution exposure on the prognosis of STEMI.

The impacts on health due to air pollution exhibit seasonal variations. For instance, Ishii et al. found a positive correlation between exposure to PM_2.5_, and the risk of MI, with the risk being more pronounced in the spring season [13], and children were more susceptible to NO_2_ on pneumonia during warm days [14]. Despite growing evidence on the impact of air pollution on health outcomes, its specific effects on the short-term outcomes of STEMI and potential seasonal effects remain unclear. Therefore, the objective of this study was to investigate the association between air pollution, weather conditions, and short-term outcomes of STEMI to better understand the impact of air pollution on STEMI prognosis and its potential seasonal effect.

## 2. Materials and Methods 

### 2.1. Study Population

This retrospective observational study was performed at an urban tertiary medical center in Kaohsiung, Taiwan, with an annual average of 72,000 ED visits and 2500 beds, spanning from 1 January 2012 to 31 December 2017. For this study, medical records of non-trauma adult patients who were over 20 years of age and visited the emergency department with a primary diagnosis of STEMI (International Classification of Diseases, Ninth Revision [ICD-9]: 410; ICD-10: I21.0–I21.3), and subsequently underwent percutaneous coronary intervention (PCI), were enrolled. Both the ED physicians and cardiologists confirmed the diagnosis of STEMI. Patient information, including age, sex, and STEMI predictive variables such as hypertension, diabetes, current smoking status, Killip classification, body mass index, and clinical outcomes, were obtained from their medical records. 

### 2.2. Pollutant and Meteorological Data

**Figure 1:**
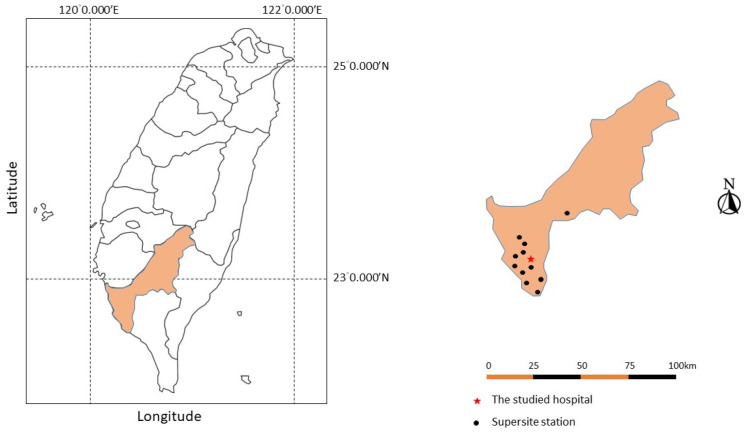
The locations of 11 air-quality monitoring stations established in Kaohsiung City. Note: the Taiwan map outline was adapted from https://webvectormaps.com/taiwan-map-outline-free-blank-vector-map/ (accessed on 29 May 2023), which was licensed under the Creative Commons Attribution 4.0 International License.

This study used air pollution data and weather conditions collected from 11 air-quality monitoring stations established in Kaohsiung City. These monitoring stations were set up by the Taiwanese Environmental Protection Administration in 1994, as shown in Figure 1. Kaohsiung is a city with a tropical climate and is situated in southern Taiwan at an elevation of approximately 9 m above sea level. Air pollutants were measured as described previously [15]. Briefly, the monitoring stations employed commercial monitoring instruments manufactured by Thermo Environmental Instruments, Inc. (Franklin, MA, USA) and designated by the United States Environmental Protection Agency (US EPA). The monitoring stations utilized full automation and monitored “criteria” pollutants on an hourly basis, which included particulate matter, PM_10_, PM_2.5_ (measured by beta-ray absorption), nitrogen dioxide (NO_2_) (measured by ultraviolet fluorescence), and ozone (O_3_) (measured by ultraviolet photometry).

The addresses of the patients were gathered from their medical files, and the 24 h average levels of these pollutants, along with the temperature and mean humidity from the monitoring station in closest proximity, were documented. The air pollutant concentration and meteorological data recorded on the same day as the patient’s ED visit were identified as a lag of 0. The values recorded on the day prior to the patient’s ED visit were identified as lag 1, and so forth. The mean concentration from lags 0 to 3 was categorized as lag 0–3. 

### 2.3. Variables and Outcome Measures 

Data on age, sex, triage status, and prognostic factors for STEMI, including comorbidities such as hypertension, dyslipidemia, diabetes, and coronary artery disease, were collected from medical records. The outcome measurement in this study was in-hospital mortality, defined as death occurring during the current hospitalization and attributable to the STEMI episode. Each episode of STEMI was considered an individual event in this study. 

This study was approved by the Institutional Review Board of the Chang Gung Memorial Hospital (number: 202101652B0C503) and was conducted in accordance with the Code of Ethics of the World Medical Association (Declaration of Helsinki).

### 2.4. Statistical Analyses

The independent variables were analyzed descriptively and presented as percentages or means ± standard deviations (SDs). The relationships between the independent variables and admission were evaluated using χ^2^, Mann–Whitney U, and Student t tests. We utilized logistic regression analysis to examine the statistical significance of the association between air pollutants, comorbidities, and the outcome of STEMI, and to calculate the odds ratio (OR), 95% confidence interval (CI), and *p* value. In order to assess whether there is a dose-dependent effect of air pollution on the prognosis of STEMI, we categorized air pollutants into different quartiles and performed logistic regression analysis to calculate the impact of STEMI prognosis at different concentrations of air pollutants. Statistical significance was set at *p <* 0.05. All statistical analyses were conducted utilizing SPSS version 25.0 (IBM Corp., Armonk, NY, USA).

## 3. Results

Over the course of the six-year study period, 1153 events met the inclusion criteria, of which 132 patients were excluded because their addresses were not in Kaohsiung City, and 18 patients were omitted due to being transferred to other hospitals or being discharged against medical advice. The final study population consisted of 1003 STEMI episodes. Among the 1003 STEMI events included in the study, four of them were repeat episodes. The demographic characteristics and air pollution conditions are presented in Table 1. Of the 1003 patients included in this study, 56 (5.6%) died during hospitalization. Most patients who survived until hospital discharge were current smokers (*p <* 0.001), had a higher frequency of dyslipidemia (*p =* 0.008), and had lower Killip classification levels (*p <* 0.001). Patients who died during hospitalization had higher NO_2_ exposures in lags 2 (*p =* 0.017), 3 (*p =* 0.005), and lag 0–3 (*p =* 0.027). 

### 3.1. Air Pollutants and Meteorological Results

Table 2 summarizes the meteorological factors, daily mean concentrations of air pollutants, and weather variables in Kaohsiung during the study period. The average concentrations of PM_2.5_, PM_10_, NO_2_, and O_3_ during the study period were 31.3 μg/m^3^, 63.5 μg/m^3^, 17.1 ppb, and 29.0 ppb, respectively. Seasonal variations in the concentrations of air pollutants were observed between the cold season (October to March) and warm season (April to September). Statistical analysis revealed that PM_2.5_, PM_10_, NO_2_, SO_2_, and O_3_ levels were significantly higher during the cold season (*p <* 0.001). Conversely, temperature and humidity levels were significantly lower during the cold season (*p <* 0.001).

### 3.2. Association between Air Pollutants Exposure and In-Hospital Mortality for STEMI

**Figure 2:**
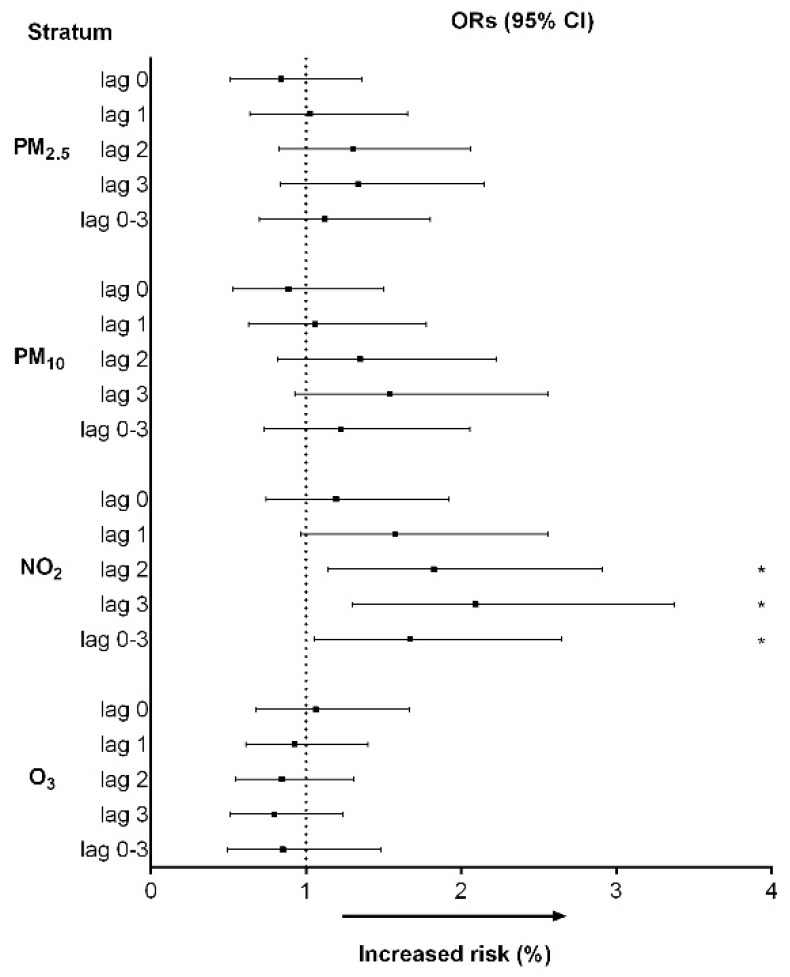
Multivariable odds ratios (with 95%CIs) for in-hospital mortality per IQR increase in air pollutants after adjusting for current smoker, dyslipidemia, Killip III–IV, temperature, and humidity. * *p <* 0.05.

A binary logistic regression model was employed to investigate the relationship between air pollutant exposure and the risk of in-hospital mortality due to STEMI. As shown in Figure 2, after adjusting for current smoker, dyslipidemia, Killip classification, and meteorological factors such as temperature and humidity, the interquartile range (IQR) increments of NO_2_ were significantly associated with the risk of in-hospital mortality in lag 2 (OR: 1.824, 95%CI: 1.142–2.313, *p =* 0.012), lag 3 (OR:2.093, 95%CI: 1.299–3.371, *p =* 0.002), and lag 0–3 (OR: 1.670, 95%CI: 1.054–2.646, *p =* 0.029). 

**Figure 3:**
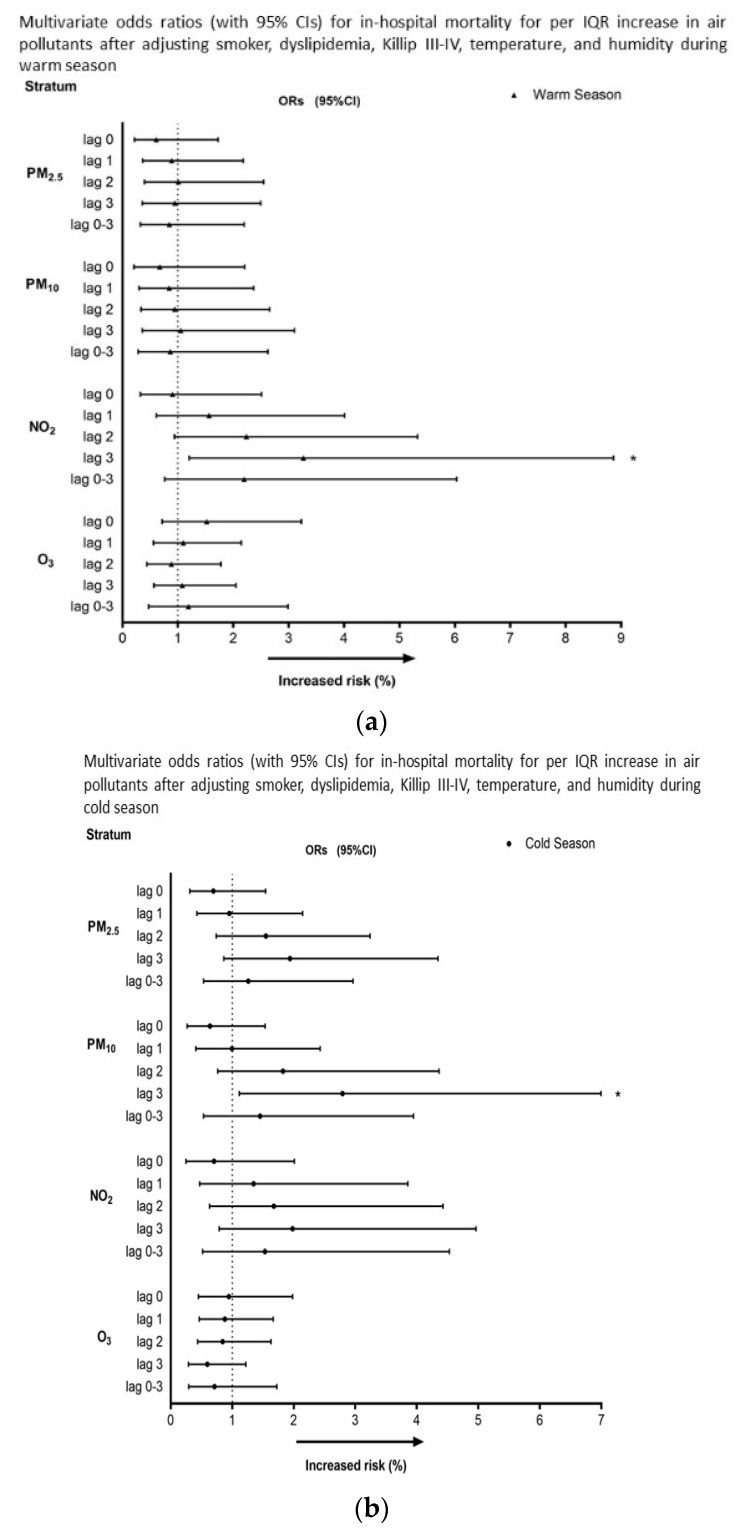
Multivariate odds ratios (with 95%CIs) for in-hospital mortality per IQR increase in PM_2.5_, PM_10_, and NO_2_ during the (**a**) warm season and (**b**) cold season. Adjustments were made for current smoker, dyslipidemia, Killip III–IV, temperature, and humidity. * *p <* 0.05.

To clarify the seasonal effect of each air pollutant on STEMI outcome, a binary logistic regression model was conducted according to the warm season (April to September) and cold season (October to March). As shown in Figure 3, during the warm season, NO_2_ was significantly associated with the risk of in-hospital mortality in lag 3 (OR: 3.266, 95%CI: 1.203–8.864, *p =* 0.02); however, the effect of NO_2_ was not statistically significant during the cold season. During the cold season, PM_10_ demonstrated a significant correlation with the risk of in-hospital mortality in lag 3 (OR:2.792, 95%CI:1.115–6.993, *p =* 0.028); however, the effect of PM_10_ was not statistically significant during the warm season.

**Figure 4:**
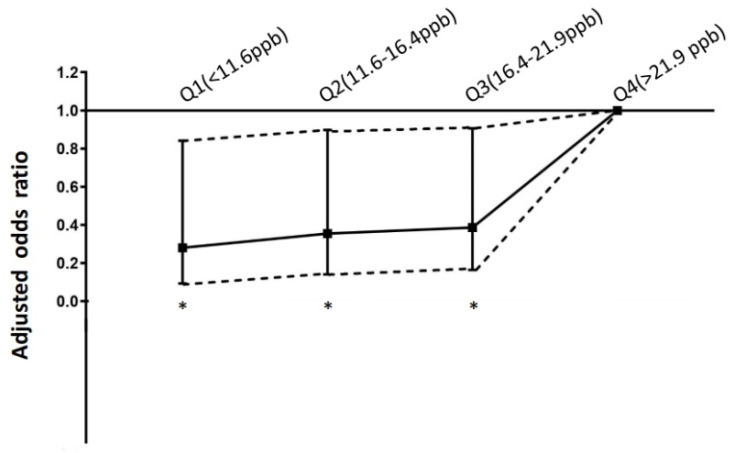
Relationship between ambient NO_2_ levels and adjusted risk for in-hospital mortality of ST-segment elevation myocardial infarction. The y-axis represents the odds ratio with 95% confidence intervals. * *p <* 0.05.

The exposure–response relationship between NO_2_ levels and the risk of STEMI was calculated to explore the potential threshold. Figure 4 shows that decreased levels of NO_2_ were significantly associated with a decreased risk of in-hospital mortality compared to higher levels of NO_2_ (Q4, >21.9 ppb). Compared to Q4 level NO_2_, exposure to Q1 level (NO_2_ < 11.6 ppb), Q2 level (NO_2_ 16.4–16.4 ppb), and Q3 level (NO_2_ 16.421.9 ppb) were significantly associated with a decreased risk for in-hospital mortality, and the ORs (95%CIs) were 0.280 (0.093–0.842, *p =* 0.023), 0.355 (0.140–0.898, *p =* 0.029), and 0.386 (0.164–0.906, *p =* 0.029), respectively. 

## 4. Discussion

In the present study, we estimated the effect of air pollution on the short-term prognosis of STEMI in Kaohsiung, Taiwan. Among all air pollutant exposures examined in this analysis, higher NO_2_ exposure levels were linked to an elevated risk of in-hospital mortality in patients with STEMI, especially during the warm season. In contrast, higher PM_10_ exposure levels were linked to an elevated risk of in-hospital mortality in patients with STEMI during the cold season.

Several epidemiological studies have revealed the detrimental effects of air pollution on MI. Bañeras et al. conducted a population-based study that included all STEMIs in Barcelona and found that PM_2.5_, PM_10_, and NO_2_ were positively associated with the incidence of STEMI [2]. In contrast, a separate study that investigated the relationship between air pollution and acute coronary syndrome found that NO_2_ exposure was positively associated with STEMI incidence, whereas the association between PM_2.5_ and PM_10_ exposure and STEMI did not reach statistical significance [16]. A recent article reviewed 56 studies and concluded that PM_2.5_, PM_10_, and NO_2_ were related to an increased risk of hypertension and subsequent MI [5]. Nevertheless, limited attention has been given to the relationship between short-term outcomes of STEMI and air pollution. In the current study, NO_2_ was positively associated with the risk of in-hospital mortality in patients with STEMI, especially during the warm season, and PM_10_ exposure levels were associated with an increased risk of in-hospital mortality in patients with STEMI during the cold season. Numerous toxicological studies have attempted to elucidate the mechanisms underlying health hazards caused by air pollution. In terms of pulmonary toxicity, cell-based studies have shown that exposure to PM activates the nuclear factor kappa-light-chain-enhancer of activated B cells (NF-κB) and triggers the NF-κB-mediated inflammatory response, leading to an increase in inflammatory cytokines, including interleukin (IL)-6, IL-8, and IL-1β in human tracheal epithelial cells [17], while animal experiments have demonstrated that exposure to PM causes infiltration of inflammatory cells in the lungs, thickening of the tracheal epithelium, and alveolar rupture [8]. These inflammatory substances include cytokines, activated immune cells, and factors that induce vascular activity, such as endotoxins, histamine, and microparticles, which are involved in the inflammatory response and enter extrapulmonary organs through the bloodstream [18,19]. In addition, exposure to NO_2_ has also been found to increase the levels of inflammation markers in the blood, including C-reactive protein (CRP), tumor necrosis factor-α, IL-6, and coagulation-related factors such as fibrinogen, as well as tissue repair marker hepatocyte growth factor [20]. These inflammatory cytokines and coagulation-related factors may cause vasoconstriction and affect clot formation in vascular endothelial cells [21]. In contrast, ultrafine particles (UFP) and certain components of PM, such as organic compounds and heavy metals, may directly penetrate the alveolar and capillary barriers of the lungs, enter the systemic circulation, and induce vascular injury [22,23]. Furthermore, while causing inflammation in the lungs, the interaction between air pollutants and lung receptors can lead to reflex responses in the autonomic nervous system, resulting in an increased heart rate, vasoconstriction, and other reactions [24,25]. Increased heart rate, vasoconstriction, disturbances in vascular endothelial clot formation, and coagulation biomarkers may affect the outcomes of MI. Animal studies have shown that exposure to NO_2_ can interfere with the regulation of endothelial nitric oxide synthase and intercellular adhesion molecule 1 in vascular endothelial cells, whereas exposure to PM_2.5_ has been found to interfere with the regulation of the renin–angiotensin system, which regulates blood pressure, possibly leading to increased blood pressure and enhanced coagulation responses [26,27]. 

The effects of air pollution on human health seemed to vary seasonally. For example, Hsu et al. found a positive correlation between PM_2.5_, concentration, and hospitalization for cardiovascular diseases, especially during the winter season [28], while Huang et al. found a correlation between elemental carbon in PM_2.5_ and the risk of chronic obstructive pulmonary disease (COPD) ED visits, especially during the warm season [29]. This can be attributed to several factors. First, the sources and composition of PM pollution particles vary across seasons, which may result in different health hazards. For example, PM_2.5_, measured at roadside locations, contains high levels of metal components, such as copper, zinc, iron, and calcium, from vehicle emissions and road dust, which are more than twice the levels found in urban background locations [30]. On the other hand, in Beijing, the concentration of PM_2.5_-bound polycyclic aromatic hydrocarbons (PAHs) is higher during the winter season compared to the summer season [31]. The total amount of PAHs during winter may be up to three times higher than in summer [32]. This difference is likely due to increased coal combustion for heating purposes during the winter. Furthermore, there are variations in the composition of metal components in PM_2.5_ during different seasons. Qi et al. conducted an analysis of PM_2.5_ collected in Suburban Nanjing and found that Aluminum and Cadmium exhibited higher concentrations during the summer, while Chromium, Nickel, and Arsenic showed higher concentrations during the winter [33]. PM is composed of particles of different sizes and chemical characteristics, and its health effects may differ depending on the composition of the components. Altemose et al. collected PM_2.5_ data before, during, and after the 2008 Beijing Olympics and measured coagulation-related biomarkers in the plasma of 128 volunteers as well as oxidative stress indicators in their exhaled breath. The results showed that PM_2.5_, generated by automobiles, factories, and biomass burning, is positively associated with lung inflammation-related biomarkers. The increase in oxidative stress was related to emissions from factories and vehicles, while coagulation-related biomarkers, such as the von Willebrand Factor (vWF), were positively associated with the combustion of fossil fuels [34]. Hwang et al. obtained data from Taiwan’s National Health Insurance program and found that PM_2.5_ was positively associated with asthma ED visits, especially for nitrate (NO_3_^−^) of PM_2.5_ [35]. Toxicological evidence also suggests that exposure to water-soluble extracts of PM_2.5_ could cause a proliferative response in the livers of mice, while insoluble particles can cause an inflammatory response and an increase in apoptosis regulation in the livers of mice [36]. Secondly, different PM components in particulate matter may interact with gaseous pollutants, resulting in different health risks. For instance, the interaction between sulfate and nitrate in PM_2.5_ and ozone (O_3_) may increase the risk of pediatric pneumonia emergency department visits [37]. Third, changes in temperature may have an additive effect on health hazards caused by air pollution. For instance, Imaizumi et al. reported a positive association between exposure to PM_2.5_ and morning hypertension and noted that this effect was strengthened by low temperatures [38]. Epidemiological studies have also found that exposure to PM_2.5_ increases the risk of asthma attacks in children, particularly on cold days [1]. Hsu et al. analyzed climate and air pollution data in New York State and examined their impact on cardiovascular hospitalization risk. The results revealed that PM_2.5_ increases the risk of cardiovascular hospitalization, especially in colder temperatures [28].

### Limitations

Several limitations were identified in this study. First, it was conducted at a single hospital, and the sample size was limited. Second, this study was conducted in tropical areas, which may limit the generalizability of the results to other areas with different ethnic and meteorological characteristics. Additionally, personal exposure may be influenced by factors such as air-conditioning usage and time spent outdoors, which may impact the observed associations compared to other geographical locations. Lastly, our study only included patients who sought hospital treatment for STEMI. Some patients may experience STEMI as an out-of-hospital cardiac arrest (OHCA) and would not be captured in our database.

## 5. Conclusions

The present study collected data on STEMI patients and air pollution, as well as climate information, spanning a period of six years. The aim was to analyze the impact of air pollution exposure on the prognosis of STEMI. To summarize, our study indicates that exposure to NO_2_ and PM_10_ may increase the risk of poor prognosis in patients with STEMI. It is worth noting that the effects of NO_2_ were more pronounced during the warm season, while the effects of PM_10_ were more significant during the cold season. The findings may suggest the need for continued efforts in reducing air pollution and stricter regulation of air quality.

## Figures and Tables

**Table 1:** Demographic characteristics, Killip classification, and air pollution conditions (mean ± standard deviation, SD) of 1003 patients with ST-segment elevation myocardial infarction (STEMI).

	Survival to Discharge	In-Hospital Mortality	
Characteristics	*N* = 947	*N* = 56	*p*
Male	789	46	0.819
Age	60.3 ± 12.7	60.1 ± 12.9	0.652
Diabetes	359	24	0.459
Hypertension	595	34	0.75
Current smoker	531	17	<0.001 ***
Dyslipidemia	696	32	0.008 **
Killip III to IV	193	38	<0.001 ***
Body mass index	25.4 ± 3.7	24.6 ± 5.1	0.192
History of coronary artery disease	53	4	0.627
PM_2.5_, μg/m^3^			
lag 0	34.3 ± 19.6	31.9 ± 18.3	0.363
lag 1	33.9 ± 19.7	34.1 ± 17.7	0.954
lag 2	33.6 ± 19.2	36.6 ± 19.6	0.261
lag 3	33.7 ± 19.0	36.2 ± 21.3	0.345
lag 0–3	33.8 ± 17.7	34.7 ± 17.7	0.724
PM_10_, μg/m^3^			
lag 0	65.3 ± 29.8	63.1 ± 28.5	0.595
lag 1	65.0 ± 30.1	66.2 ± 26.7	0.766
lag 2	64.8 ± 29.3	69.8 ± 30.3	0.217
lag 3	64.8 ± 29.2	70.7 ± 32.9	0.145
lag 0–3	65.2 ± 26.9	67.9 ± 26.9	0.456
NO_2_, ppb			
lag 0	17.7 ± 6.5	18.7 ± 7.3	0.252
lag 1	17.6 ± 6.6	19.3 ± 7.1	0.075
lag 2	17.6 ± 6.7	19.9 ± 7.9	0.017 *
lag 3	17.8 ± 6.7	20.4 ± 7.8	0.005 **
lag 0–3	17.7 ± 6.1	19.6 ± 7.0	0.027 *
O_3_, ppb			
lag 0	28.5 ± 12.2	28.3 ± 12.2	0.883
lag 1	28.6 ± 12.7	27.9 ± 14.8	0.677
lag 2	28.2 ± 12.5	27.1 ± 11.8	0.5
lag 3	28.3 ± 12.7	26.8 ± 11.4	0.403
lag 0–3	28.4 ± 10.7	27.5 ± 10.4	0.543

* *p <* 0.05, ** *p <* 0.01. *** *p <* 0.001.

**Table 2:** Summary statistics for air pollution and meteorology in Kaohsiung, 2012–2017.

	Minimum	Percentiles			Maximum	Mean	Warm Season (Mean ± SD)	Cold Season (Mean ± SD)	*p*
		25%	50%	75%					
PM_2.5_ μg/m^3^	1.6	16.1	29.9	44.1	120.8	31.3 ± 17.8	16.9 ± 11.9	42.9 ± 14.9	<0.001
PM_10_ μg/m^3^	16.1	37.0	61.0	84.7	181.0	63.5 ± 28.8	43.1 ± 17.0	84.0 ± 23.2	<0.001
NO_2_ (ppb)	4.8	11.6	16.4	21.9	35.0	17.1 ± 7.4	12.6 ± 6.6	21.7 ± 5.0	<0.001
O_3_ (ppb)	3.5	18.6	27.1	36.6	61.7	28.4 ± 12.4	23.2 ± 13.2	30.7 ± 11.2	<0.001
Temperature (°C)	7.1	22.5	26.5	29.0	32.1	25.5 ± 4.2	28.5 ± 1.9	22.5 ± 3.6	<0.001
Humidity (%)	35.3	70.4	73.8	77.4	94.4	74.0 ± 6.6	75.4 ± 6.4	72.5 ± 6.6	<0.001

## Data Availability

The datasets used and analyzed during the current study are available from the corresponding author upon reasonable request.
